# Correction: Stafseth et al. Symptoms of Anxiety, Depression, and Post-Traumatic Stress Disorder in Health Care Personnel in Norwegian ICUs during the First Wave of the COVID-19 Pandemic, a Prospective, Observational Cross-Sectional Study. *Int. J. Environ. Res. Public Health* 2022, *19,* 7010

**DOI:** 10.3390/ijerph192315424

**Published:** 2022-11-22

**Authors:** Siv Karlsson Stafseth, Laila Skogstad, Johan Ræder, Ingvild Strand Hovland, Haakon Hovde, Øivind Ekeberg, Irene Lie

**Affiliations:** 1 Department of Postoperative and Intensive Care, Division of Emergencies and Critical Care, Oslo University Hospital, 0424 Oslo, Norway; 2 MEVU, Lovisenberg Diaconal University College, 0456 Oslo, Norway; 3 Department of Health and Care Sciences, Faculty of Health Sciences, UiT The Arctic University of Norway, 9019 Tromsø, Norway; 4 Department of Anaesthesiology, Division of Emergencies and Critical Care, Oslo University Hospital, 0424 Oslo, Norway; 5 Institute of Clinical Medicine, University of Oslo, 0313 Oslo, Norway; 6 Department of Acute Medicine, Division of Medicine, Oslo University Hospital, 0424 Oslo, Norway; 7 The Norwegian Association for Critical Care Nurses, 0152 Oslo, Norway; 8 Psychosomatic and Consultation-Liaison Psychiatry, Division of Mental Health and Addiction, Oslo University Hospital, 0424 Oslo, Norway; 9 Centre for Patient-Centered Heart and Lung Research, Department of Cardiothoracic Surgery, Division of Cardiovascular and Pulmonary Diseases, Oslo University Hospital, 0424 Oslo, Norway; 10 Department of Health Sciences in Gjøvik, Faculty of Medicine and Health Sciences, Norwegian University of Science and Technology, 2815 Gjøvik, Norway

**Error in Table**

In the original publication [1], there was a mistake in Table 2. Symptoms of anxiety and depression in health care professionals using the Hopkins Symptom Checklist-10 (HSCL-10) during the past week and symptom-defined PTSD (PCL-5) during the past month (N = 484) as published. For HSCL-10 total score, Mean (SD) and CI 95% has been corrected for RNs and Leaders, and for PCL-5 total score Mean (SD) and CI 95%, Range (min–max) has been corrected for all three groups: RNs, Physicians and Leaders. The corrected Table 2. Symptoms of anxiety and depression in health care professionals using the Hopkins Symptom Checklist-10 (HSCL-10) during the past week and symptom-defined PTSD (PCL-5) during the past month (N = 484) appears below. The authors state that the scientific conclusions are unaffected. This correction was approved by the Academic Editor. The original publication has also been updated.

## Figures and Tables

**Table 2:** Symptoms of anxiety and depression in health care professionals using the Hopkins Symptom Checklist-10 (HSCL-10) during the past week and symptom-defined PTSD (PCL-5) during the past month (N = 484).

	RNs (*n* = 392)	Physicians (*n* = 43)	Leaders (*n* = 49)	*p*
Anxiety and depression HSCL-10				
Symptoms of anxiety * Mean (SD) Symptoms of depression * Mean (SD) HSCL-10 total score, Mean (SD) CI 95% Range (min–max)	1.31 (0.48) 1.41 (0.50) 1.36 (0.46) 1.31–1.41 (1.0–3.6)	1.20 (0.40) 1.31 (0.46) 1.27 (0.42) 1.14–1.40 (1.0–2.5)	1.22 (0.29) 1.27 (0.32) 1.29 (0.33) 1.19–1.38 (1.0–2.2)	0.17 0.13 0.098
Symptoms of anxiety *n* (%) Symptoms of depression *n* (%) No. total symptoms HSCL-10 (%)	42 (10.71) 51 (13.01) 49 (12.5)	5 (11.63) 4 (9.30) 5 (11.63)	2 (4.08) 2 (4.08) 2 (4.08)	0.098
PTSD (PCL-5)				
PCL-5 total score ** Mean (SD) CI 95% Range (min–max)	10.2 (11.02) 9.11–11.30 (0–64)	5.2 (8.16) 2.70–7.72 (0–36)	7.94 (8.23) 5.57–10.30 (0–35)	<0.001 ***
No. total symptom-defined PTSD (%)	28 (7.14)	1 (2.32)	2 (4.08)	<0.001 ***

Note: * Scale HSCL-10 (1 = Not bothered to 4 = Very much bothered), cut-off score of ≥1.85, ** Scale PCL-5 (0 = Not at all to 4 = Extremely) cut-off score of ≥31, CI = confidence intervals; SD = standard deviation, Kruskal–Wallis test used between groups, *** pair-wise comparison with Bonferroni correction detected no difference between groups tests.
