# Supplementary material for: Symptoms of Anxiety, Depression, and Post-Traumatic Stress Disorder in Health Care Personnel in Norwegian ICUs during the First Wave of the COVID-19 Pandemic, a Prospective, Observational Cross-Sectional Study
Source: Int J Environ Res Public Health. 2022 Jun 8;19(12):7010. doi: 10.3390/ijerph19127010 (PMC9222786; doi:10.3390/ijerph19127010)
Supplement: Supplementary file 1 [file ijerph-19-07010-s001.zip › ijerph-1732152-supplementary.pdf]

International Journal of
*Environmental Research and Public Health*

MDPI

*Article*

# Symptoms of Anxiety, Depression, and Post-Traumatic Stress Disorder in Health Care Personnel in Norwegian ICUs during the First Wave of the COVID-19 Pandemic, a Prospective, Observational Cross-Sectional Study

**Siv K Stafseth[1,2*], Laila Skogstad[3], Johan Raeder[4,5], Ingvild Strand Hovland[6], Haakon Hovde[7], Øivind Ekeberg[8] and Irene Lie[9,10]**

Department of Postoperative and Intensive care, Division of Emergencies and Critical Care, Oslo University Hospital, Oslo, Norway

2 Lovisenberg Diaconal University College, Oslo, Norway

3 Department of Health and Care Sciences, Faculty of Health Sciences, UiT The Arctic University of Norway, Tromsø, Norway

4 Department of Anaesthesiology, Division of Emergencies and Critical Care, Oslo University Hospital, Oslo, Norway

5 Institute of Clinical Medicine, University of Oslo, Norway

6 Department of Acute medicine, Division of Medicine, Oslo University Hospital, Oslo, Norway

7 The Norwegian Association for critical care nurses, Oslo, Norway

8 Psychosomatic and Consultation-Liaison psychiatry, Division of Mental Health and Addiction, Oslo University Hospital, Oslo, Norway

9 Centre for Patient-Centered Heart and Lung Research, Department of Cardiothoracic surgery, Division of Cardiovascular and Pulmonary Diseases, Oslo University Hospital, Oslo, Norway

10 Department of Health Sciences in Gjøvik, Faculty of Medicine and Health Sciences, Norwegian University of Science and Technology, Gjøvik, Norway

* Correspondence: sistaf@ous-hf.no

**Questionaire PART IV-VII**

**Healthcare professionals' experiences with COVID-19 patients in intensive care units in Norway (Part IV-VII)**

Reference ID

- The field is filled in automatically

Thank you for taking the time to share your views and experiences working with COVID-ICU patients.

**Part IV: CORONAVIRUS AND SOCIAL CONTACT**

Who have you been afraid of infecting?

*Choose all that apply*

- o Patients
- o Colleagues
- o Family in own household or other family members
- o Friends
- o Not applicable

How would you describe your limited social contact?

| | Not at all | To a small degree | Partly | To a high degree | To a very high degree |
|---|---|---|---|---|---|
| Felt isolated | o | o | o | o | o |
| Felt lonely | o | o | o | o | o |
| No problem | o | o | o | o | o |
| Increased contact within own household | o | o | o | o | o |
| Increased contact via social media, telephone, work | o | o | o | o | o |

How have you had social contact outside working hours?

*Choose all that apply.*

- o Social media like Facebook
- o Talked to colleague(s) by phone
- o Communicated with friends, family on SKYPE, Facetime, etc.
- o Met with others, but only outdoors with social distancing and masks
- o Met with a limited number of people indoors with masks and social distancing
- o Met with others indoors, one to several times beyond recommendation from the Norwegian Institute of Public Health (NIPH) (number of people, social distancing)

## Part V: ASSESSMENT OF INDIVIDUAL WORK EFFORT AND SUPPORT MEASURES

To what extent are you professionally satisfied with the following aspects of your work?

| | Not at all | To a small degree | Partly | To a high degree | To a very high degree |
|---|---|---|---|---|---|
| Your own effort | o | o | o | o | o |
| Your closest partners | o | o | o | o | o |
| Your department | o | o | o | o | o |
| Your organization | o | o | o | o | o |
| Responsible authorities | o | o | o | o | o |

Assessment of proficiency during work in the COVID-ICU

| | Not at all | To a small degree | Partly | To a high degree | To a very high degree |
|---|---|---|---|---|---|
| To what extent do you think that the work you participate / participated in was successful? | o | o | o | o | o |

| | | | | | |
|---|---|---|---|---|---|
| To what extent do you think that the work you participate / participated in was meaningful? | o | o | o | o | o |
| To what extent do / did you experience falling short of the work tasks to be performed? | o | o | o | o | o |
| To what extent do / did you experience that you were impeded in the work you or your team had to perform (e.g. due to bureaucracy, lack of equipment, unclear messages?) | o | o | o | o | o |
| Did you receive sufficient advice and support at work? | o | o | o | o | o |
| Did you overextend yourself because great effort was expected of your team, leader or colleagues (e.g. did not take breaks, worked more than you really could, etc.)? | o | o | o | o | o |

Recognition and support during the pandemic

| | **Not at all** | **To a small degree** | **Partly** | **To a high degree** | **To a very high degree** |
|---|---|---|---|---|---|
| To what extent have your efforts been appreciated by your hospital? | o | o | o | o | o |
| To what extent have your family / friends given you recognition for your professional and personal efforts? | o | o | o | o | o |
| To what extent have you experienced that the authorities have recognized the work your professional group has done during the pandemic? | o | o | o | o | o |

Does / did your employer have any kinds of support measures in place during the COVID-ICU effort?

*Choose all that apply*

- o No special support measures
- o Yes, group meetings where experiences could be shared (debriefing, defusing, etc.)
- o Yes, professional support measure (psychologist, psychiatrist, psychiatric nurse, chaplain, minister, etc.)
- o Information about support measures was provided
- o I declined support talks
- o Support by phone (e.g. from work environment)
- o Follow-up by manager following completed shifts

- Other

Are there other elements at work that have seemed supportive? (Choose all that apply.)

- Talking to a colleague
- Talking to a manager
- Professional information
- Daily briefing
- Not applicable

Have you considered quitting your job as a result of the pandemic?

- No
- Yes, sometimes
- Yes, often
- Yes, specifically planning to quit / have already resigned

**Part VI: REACTIONS TO EXPERIENCES AND ECONOMY**

Below is a list of issues that may be reactions to highly stressful events such as the COVID-19 pandemic.

*Please indicate whether any experiences related to the COVID-19 pandemic have been stressful for you by answering each question.*

*Have you experienced any of the following issues during the last month?*

| | Not at all | A little bit | Moderately | Quite a bit | Extremely |
|---|---|---|---|---|---|
| Repeated, disturbing, and unwanted memories of the stressful experiences of the pandemic? | o | o | o | o | o |
| Repeated, disturbing dreams of the stressful experience of the pandemic? | o | o | o | o | o |
| No problemSuddenly feeling or acting as if the stressful experience were actually happening again ( as if you were actually back there reliving it)? | o | o | o | o | o |
| Feeling very upset when something reminded you of the stressful experience of the pandemic? | o | o | o | o | o |
| Having strong physical reactions when something reminded you of the stressful experience of the pandemic? | o | o | o | o | o |
| Avoiding memories, thoughts, or feelings related to the stressful experience of the pandemic? | o | o | o | o | o |
| Avoiding external reminders of the stressful experience of the pandemic (for example, people, places, conversations, activities, objects, or situations)? | o | o | o | o | o |

| | | | | | |
|---|---|---|---|---|---|
| Trouble remembering important parts of the stressful experience of the pandemic? | ○ | ○ | ○ | ○ | ○ |
| Having strong negative beliefs about yourself, other people, or the world ( for example, having thoughts such as: I am bad, there is something seriously wrong with me, no one can be trusted, the world is completely dangerous)? | ○ | ○ | ○ | ○ | ○ |
| Blaming yourself or someone else for the pandemic? | ○ | ○ | ○ | ○ | ○ |
| Having strong negative feelings such as fear, horror, anger, guilt, or shame? | ○ | ○ | ○ | ○ | ○ |
| Loss of interest in activities that you used to enjoy? | ○ | ○ | ○ | ○ | ○ |
| Feeling distant or cut off from other people? | ○ | ○ | ○ | ○ | ○ |
| Trouble experiencing positive feelings (for example, being unable to feel happiness or have loving feelings for people close to you)? | ○ | ○ | ○ | ○ | ○ |
| Irritable behavior, angry outbursts, or acting aggressively? | ○ | ○ | ○ | ○ | ○ |
| Taking too many risks or doing things that could cause you harm? | ○ | ○ | ○ | ○ | ○ |
| Being "superalert" or watchful or on guard? | ○ | ○ | ○ | ○ | ○ |
| Feeling jumpy or easily startled? | ○ | ○ | ○ | ○ | ○ |
| Having difficulty concentrating? | ○ | ○ | ○ | ○ | ○ |
| Trouble falling or staying asleep? | ○ | ○ | ○ | ○ | ○ |

Have you experienced any of these during the last week (including today)?

| | **Not bothered** | **A little** | **Quite a bit** | **Very much bothered** |
|---|---|---|---|---|
| Suddenly scared for no reason | ○ | ○ | ○ | ○ |
| Feeling fearful | ○ | ○ | ○ | ○ |
| Faintness, dizziness or weakness | ○ | ○ | ○ | ○ |
| Feeling tense or keyed up | ○ | ○ | ○ | ○ |
| Blaming yourself for things | ○ | ○ | ○ | ○ |
| Difficulties in falling asleep or staying asleep | ○ | ○ | ○ | ○ |

| | | | | |
|---|---|---|---|---|
| Feeling blue | o | o | o | o |
| Feelings of worthlessness | o | o | o | o |
| Feeling everything is a effort | o | o | o | o |
| Feeling hopeless about the future | o | o | o | o |

Have you used one or more of the following medications as a result of your COVID-ICU work during the pandemic?

| | **Never** | **Sometimes** | **Weekly** | **Daily** |
|---|---|---|---|---|
| Sleep medications | o | o | o | o |
| Sedatives | o | o | o | o |
| Anxiolytic medications | o | o | o | o |
| Antidepressants | o | o | o | o |
| Other medications | o | o | o | o |

Have you used alcohol as a result of your COVID-ICU work during the pandemic?

| | **No** | **Yes, the first week** | **Yes, for more than a week** |
|---|---|---|---|
| To calm you down | o | o | o |
| As a mood enhancer | o | o | o |
| To sleep | o | o | o |
| Used more alcohol than you normally do | o | o | o |
| Used about as much alcohol as you normally do | o | o | o |
| Used less alcohol than you normally do | o | o | o |

Have you previously had symptoms of anxiety?

- o Yes
- o No

Have you previously had symptoms of depression?

- o Yes
- o No

How has the COVID-19 pandemic affected your finances?

- o Improved
- o Unchanged
- o Worsened

**Part VII: FINAL QUESTIONS**

| | Not at all | To a small degree | Partly | To a high degree | To a very high degree |
|---|---|---|---|---|---|
| To what extent are you more prepared to cope with a future pandemic? | o | o | o | o | o |
| To what extent do you better understand the reactions of others after the COVID-19 pandemic? | o | o | o | o | o |

Thank you so much for taking the time to respond to this survey! Your participation is greatly appreciated, and we are extremely grateful for your contribution to this research on the experiences of healthcare personnel working in COVID-ICUs in Norway.

You have received a copy of the consent form in your digital mailbox and can register your consent via the consent portal: portal.tsd.usit.no/

https://nettskjema.no/user/form/preview.html?id=147356#/ Page 29 of 29 rc1)

**Table S1 and Table S2, Results of univariate quantile regression analyses**

**Table S1.** Results of univariate quantile regression analysis of factors predicting probable mental health disorder (HSCL-10 ≥1.85).

| Exploratory variables Estimates (q=0.5) | Coefficient | Standard Error | 95%CI | t | *p* |
|---|---|---|---|---|---|
| Age<br>R 2 0.086 | -0.16 | 0.0063 | -0.028, -0.003 | -2.464 | 0.017 |
| Sex<br>R 2 0.086 | 0.003 | 0.1670 | -0.332, 0.332 | 0.019 | 0.985 |
| Married/ partner<br>Children in houshold<br>R 2 0.160 | 0.100<br>0.000 | 0.4372<br>0.4646 | -0.777, 0.977<br>-0.932, 0.932 | 0.229<br>0.000 | 0.820<br>1.000 |
| Professional Nurse<br>Physcian<br>R 2 0.006 | 0.000<br>0.100 | 0.4360<br>0.5057 | -0.874, 0.874<br>-0.914, 1.114 | 0.000<br>0.198 | 1.000<br>0.844 |
| Previous work experience (<5 years) | 0.400 | 0.1509 | 0.097, 0.703 | 2.650 | 0.011 |
| >6 years<br>R 2 0.104 | 0.200 | 0.2310 | -0.263, 0.663 | 0.866 | 0.391 |

CI=Confidence intervals, R 2=Pseudo R square.

**Table S2.** Results of univariate quantile regression analysis of symptom-defined PTSD n=31 (PCL-5 ≥31).

| Exploratory variables Estimates (q=0.5) | Coefficient | Standard Error | 95%CI | t | *p* |
|---|---|---|---|---|---|
| Age<br>R 2 0.010 | -0.063 | 0.2168 | -0.506, 0.381 | -0.288 | 0.775 |
| Sex<br>R 2 0.011 | -2.000 | 4.2836 | -10.761, 6.761 | 0.467 | 0.644 |
| Married/ partner | 4.000 | 10.1551 | -16.802, 24.802 | 0.394 | 0.697 |
| Children in household | 1.000 | 11.5147 | -22.587, 24.587 | 0.087 | 0.931 |
| R 2 0.040 | | | | | |
| Professional Nurse | -1.000 | 7.2988 | -15.951, 13.951 | -0.137 | 0.892 |
| Physcian | 1.000 | 12.2132 | -24.018, 26.018 | 0.082 | 0.935 |
| R 2 0.017 | | | | | |
| Previous work experience (<5 years) | -3.000 | 6.3069 | -15.919, 9.919 | -0.476 | 0.638 |
| >6 years | 2.000 | 3.7787 | -5.740, 9.740 | 0.529 | 0.601 |
| R 2 0.034 | | | | | |

CI=Confidence intervals, R 2=Pseudo R square.

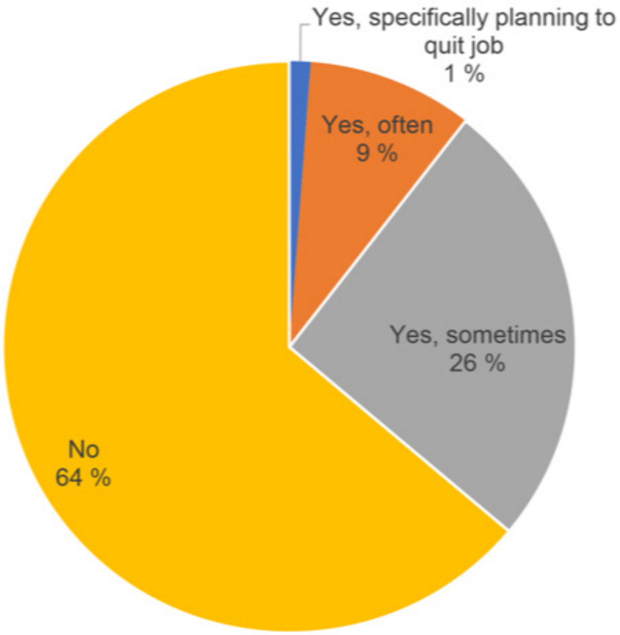


**Figure S1.** Results of the answer to “Have you considered quitting your job as a result of the pandemic?” (N=484).
